# Endoscopic stent placement for the management of gastro-pleural and gastro-cutaneous fistula post laparoscopic sleeve gastrectomy: a case report

**DOI:** 10.1186/s13256-023-04200-9

**Published:** 2023-11-06

**Authors:** Om Parkash, Zahabia Sohail, Natasha Khalid

**Affiliations:** 1 The Aga Khan University Hospital, Karachi, Pakistan; 2 Indus Hospital, Karachi, Pakistan

**Keywords:** Laparoscopic sleeve gastrectomy, Gastro-cutaneous fistula, Gastro-pleural fistula, Endoscopic stent placement

## Abstract

**Background:**

Gastro-pleural and gastro-cutaneous fistulae formation are rare yet life-threatening complications post-bariatric surgery. To our knowledge so far only limited cases of gastro-pleural and gastro-cutaneous fistulae post gastric sleeve surgery have been reported in the literature with their corresponding management. Therefore, we are reporting a case of placement of an endoscopic stent in the management of gastro-cutaneous fistula post laparoscopic sleeve gastrectomy.

**Case presentation:**

A 42 years old Pakistani, female morbidly obese patient, underwent laparoscopic sleeve gastrectomy. Within a week after the procedure, the patient presented with dyspnea. Workup showed a gastric leak for which percutaneous drain placement was done. Later, gastro-pleural and gastro-cutaneous fistulae were formed for which endoscopic fistula closure was done using a metallic stent.

**Conclusion:**

Endoscopic stent placement is an emerging field and it is considered safe and effective for the management of complications related to bariatric surgery.

## Introduction

Laparoscopic Sleeve Gastrectomy (LSG) has emerged as one of the most common bariatric procedures performed nowadays [1]. LSG is considered safe because of its technical simplicity. It requires less operative time and does limited alteration of the normal stomach anatomy [2]. Nevertheless, it has its risks. Complications of the procedure include the risk of staple-line bleeding, staple site gastric leakage, or formation of strictures [3]. Gastric leakage commonly occurs at the angle of His and is associated with increased morbidity due to fistulous tract formation and sepsis [4, 5]. The pathogenesis of gastric fistula formation is based on the theory of vascular necrosis [6]. Devascularization during LSG causes ischemia in the gastric suture line. This mechanism produces necrosis of the gastric remnant, resulting in the development of a leak which leads to the formation of an inflammatory phlegmon. The inflammatory phlegmon from the contained leak subsequently eroded through the diaphragm, setting up an inflammatory process resulting in fistulation [7, 8]. Also post LSG, a high-pressure system of a sleeved stomach from an intact pylorus distally and lower esophageal sphincter proximally, increases the risk of persistent leaks or fistulation to adjacent anatomical compartments.

The desired approach to a case with these complications should be a non-operative one, however re-operative surgery or stent insertion are the next best options once conservative measures fail [9]. With the rise in bariatric procedures, endoscopic stent placement is gaining popularity in the management of gastric leaks through the use of self-expandable metallic stents (SEMS) [10].

Gastro-pleural and gastro-cutaneous fistulae formation is an uncommon yet life-threatening complication that can occur following bariatric surgery, particularly LSG. Despite their significance, there is limited literature on their effective management. We present a unique case of successful endoscopic stent placement for the management of these complications to improve patients care and outcome. On considering a non-surgical alternative, our report adds to the growing body of evidence supporting the safety and efficacy of this approach.

## Case description

A 42-year-old morbidly obese Pakistani female with, a body mass index (BMI) of 41.8 kg/m^2^, with a recent history of sleeve gastrectomy done 2 weeks prior, presented to our hospital with persistent vomiting, abdominal pain, fever, and dyspnea. Her past medical history was significant for hypertension. She has been a full-time housewife for the past 12 years. She has not been pregnant and has not used any form of contraception. She resides in an urban area and had a sedentary lifestyle. She reports no history of smoking, drinking or illicit drug use. She had a history of emotional eating and lack of social support for weight management. No known history of exposure to environmental chemicals. The family history was significant for obesity, diabetes, and hypertension. Her medication included tablet amlodipine 5 mg and telmisartan 40 mg orally once daily. She reported to be on these medications for last 5 years.

Vital signs revealed a body temperature of 38.5 °C, heart rate of 114 beats per minute, blood pressure of 110/54 mmHg, respiratory rate of 32 breaths per minute, and oxygen saturation of 93% on room air. On examination, she was dehydrated. Chest auscultation had decreased breath sounds over the left lung base. On abdominal examination, scar marks from recent surgery were seen which were clean and dry. It was soft and non-tender with no viscera palpable. Rest of the general physical investigations and systemic investigations were unremarkable. Relevant laboratory investigations were done which are as follows; hemoglobin level 10.9 g/dl (12.3–16.6), MCV 87.6fL (78.7–96.3), white blood cell count 12.3 × 10*3/L (4.8–11.3), platelet count 507 × 10*9/L (154–433), serum blood urea nitrogen 21 mg/dl (6–20), serum creatinine 0.8 mg/dl (0.9–1.3), serum sodium 135 mmol/L (136–145), serum potassium 3.9 mmol/L (3.5–5.1), serum chloride 98 mmol/L (98–107), serum bicarbonate 25 mmol/L (20–31), PT 12.2 s (9.3–12.8), APTT 33.9 s (22.9–34.5), total bilirubin 0.4 mg/dl (0.1–1.2), GGT 41 IU/L (< 55), SGPT 18 IU/L (< 45), alkaline phosphatase 126 IU/L (45–129), AST 20 IU/L (< 35), Albumin 3.2 g/dl (3.5–5.2). Contrast-enhanced CT scan abdomen was done which showed a peri-gastric collection with an air-fluid level due to gastric fundus leakage, evident by contrast trickling from the sleeve gastrectomy site. Patient was started on intravenous (IV) hydration with 0.9% normal saline at 75 ml per hour, empiric IV antibiotic amoxicillin 1.2 g every eight hourly, IV antiemetic metoclopramide 10 mg every eight hourly. She was also administered on IV analgesic paracetamol 1 g and IV tramadol 50 mg as per need. The patient underwent CT-guided percutaneous drain placement for the abdominal collection and also left-sided pigtail catheter placement for left pleural effusion. Pleural fluid analysis was exudative. The culture of fluids from drains had bacterial growth of streptococcus species and Klebsiella pneumonia. The patient was started on susceptible antibiotic IV ciprofloxacin 400 mg every 12 hourly and continued IV amoxicillin 1.2 g every eight hourly.

**Fig. 1:**
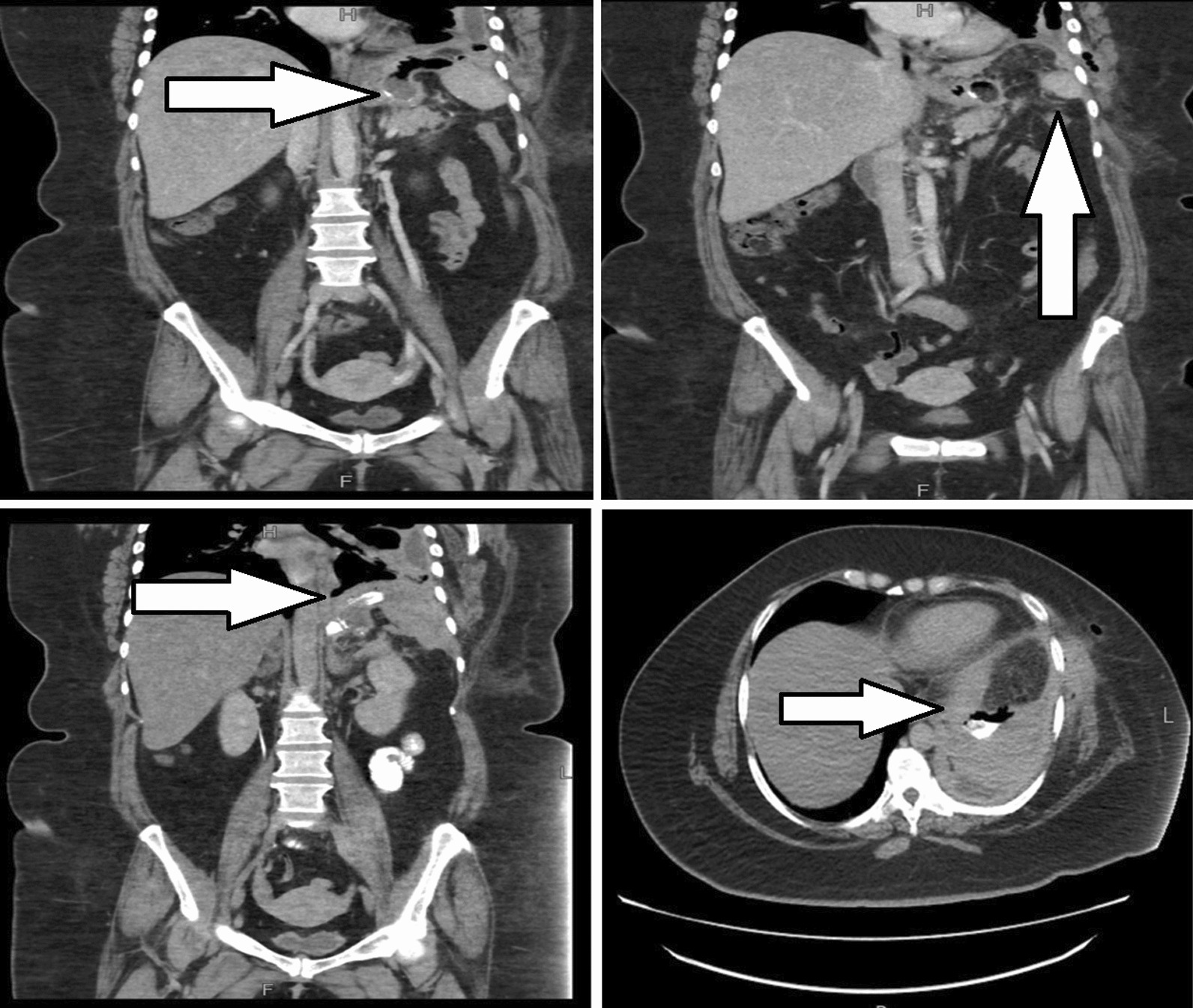
**A**–**D** CT scan of abdomen and pelvis performed with IV and oral contrast demonstrated (**A**, **B**) abnormal communication between gastric fundus, left pleural reflection over left hemi diaphragm as well as the left chest wall, representing gastro-pleural and gastro-cutaneous fistulae (arrows). This is confirmed by the administration of oral contrast (**C**, **D**). With contrast tricking from the gastric fundus into the left pleural cavity (arrows)

Due to persistent symptoms repeat contrast-enhanced CT scan was done which not only showed a fistulous tract between the collection and the skin with an external opening at the left lower chest wall but another fistulous tract was also seen communicating with the left pleural cavity resulting in empyema. These findings were suggestive of gastro-cutaneous fistula and gastro-pleural fistula formation (Fig. 1). The radiology, surgery, and gastroenterology teams' combined decisions were taken for the management of this case. For gastro-pleural fistula thoracotomy with decortication was done. Thoracoscope was inserted, and loculi were broken and drained. The drain culture had a growth of gram-positive and negative bacteria for which the patient was already on susceptible antibiotics.

**Fig. 2:**
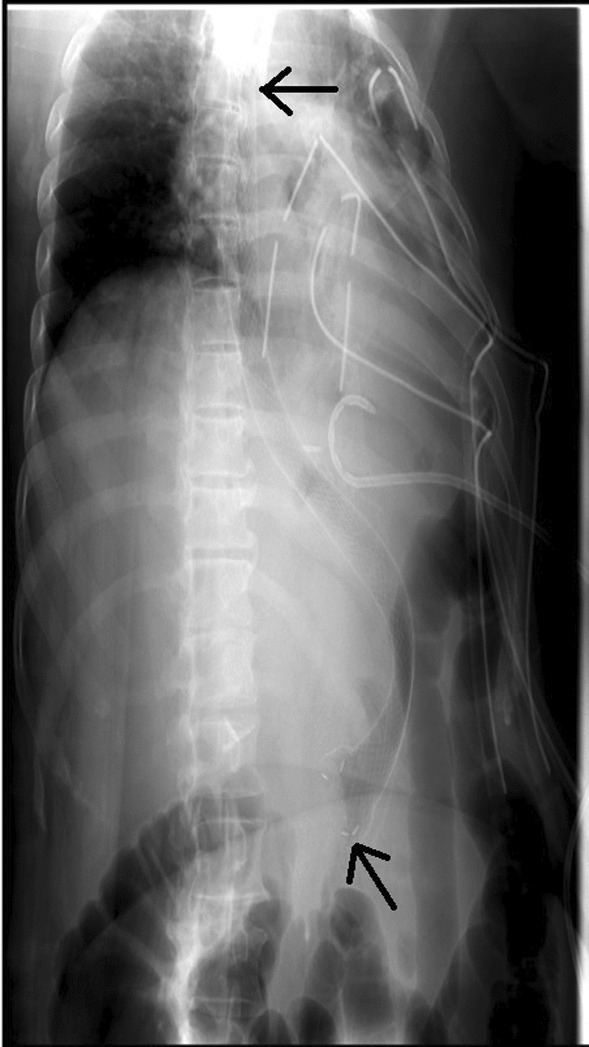
Mega, self-expanding, metallic esophageal stent fully covered with silicone (arrows) (length: 230 mm; diameter: 28 mm)

For gastro-cutaneous fistula, endoscopy and fluoroscopy-guided fistula closure were done. Gastroscope was negotiated into the stomach which was found to be long and tubular along the lesser curvature. The angle of the incisura was narrowed. Guidewire was placed in the gastric lumen through the working channel of the gastroscope. The endoscope was removed, leaving the guidewire in place, the position was confirmed with fluoroscopy. Through the guidewire, 230 mm long and 28 mm wide, mega self-expandable fully covered Niti-S metallic stent was inserted. The endoscope was reinserted and under direct visualization, the stent was opened by its delivery system and was deployed in the proximal end of the surgically repaired gastric lumen. It was extending from the mid-esophagus to the pylorus (Fig. 2). The position of the stent was confirmed via fluoroscopy.

Post-procedure, the X-ray showed no evidence of pneumo-thorax or pneumo-mediastinum. The patient was started on a liquid diet, which she tolerated well, and gradually progressed to a soft diet. She was discharged on oral medications, ciprofloxacin 400 mg twice daily, amoxicillin 625 mg thrice daily, esomeprazole 40 mg before breakfast, paracetamol 1 g and metoclopramide 10 mg for pain and vomiting respectively, as per need.

**Fig. 3:**
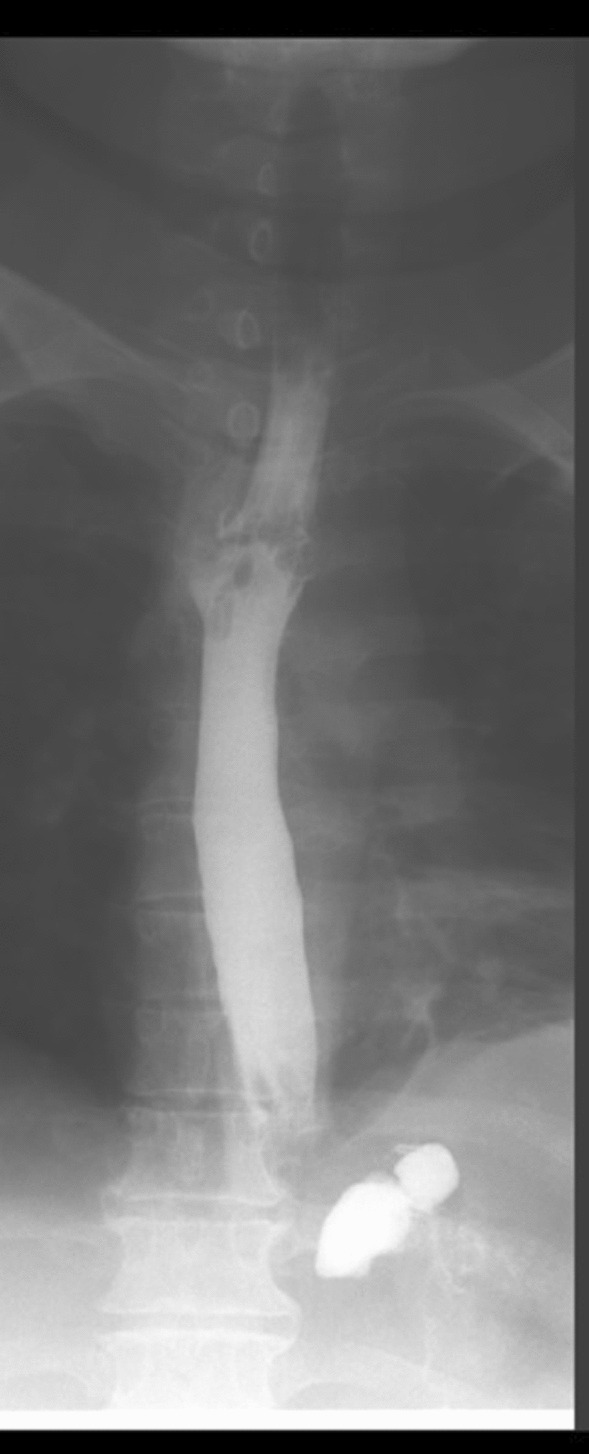
Post stent removal, upper GI contrast study is showing contrast filling esophagus and gastric fundus without residual leak into the pleural cavity, representing interval healing of fistulous communication

The patient was called for a follow-up after 6 weeks. She had a good recovery. Esophagogram was done which was negative for contrast leakage. Therefore, she underwent repeat gastroscopy for stent removal. The previously placed mega stent was seen in situ. The stent was removed successfully with the help of alligator forceps. Post-procedure no contrast leakage was seen (Fig. 3). At the time of the 6-month follow-up, the patient was doing well with no complaints. Also, she had lost 5 kg of weight from her baseline. This case report describes the successful management of gastro-cutaneous fistula post-LSG via endoscopic techniques.

## Discussion

We present a unique and complex case of an obese patient who developed gastro-pleural and gastro-cutaneous fistulae following LSG which prompted a multidisciplinary approach. In this case report, we aim to highlight the importance of endoscopy guided SEMS placement, as a viable option in managing these complex postoperative complications, ultimately contributing to improved patient care and outcomes.

Laparoscopic sleeve gastrectomy for bariatric surgery is a globally recognized procedure. Major complications include bleeding, leak, or stricture. These complications can be treated non-operatively, endoscopically, or surgically depending on severity and chronicity [11]. Gastric leakage is a serious complication post-procedure reported in around 2% of the cases [12]. Incidence of gastro-pleural fistula is rare. So far 11 cases of gastro-pleural fistula as a complication of sleeve gastrectomy have been reported in the literature [13, 14], but the incidence of gastro-cutaneous fistula following bariatric procedures is 1.7 to 4.0% [15]. One of the identified causes leading to the development of gastro-pleural fistula, which is a rare entity is sleeve gastrectomy. The symptoms of patients presenting with these issues are variable ranging from asymptomatic to symptomatic individuals presenting with cough, shortness of breath, chest pain, fever, or abdominal pain. Tachycardia is the most common sign of leaks, presenting in 72% to 92% of patients [16]. Leaked gastric content may collect next to the anastomosis or exit through the skin or the drain. Some patients are in a state of impaired hemodynamics [13].

The management has not been well standardized yet, but for both presentations, initial management is bowel rest and percutaneous drainage, as was done in our case. When conservative measures fail, an endoscopic interventional approach is an option. In a few cases, drastic measures such as surgery are required. A case report by Haider *et al.* reported one patient with a gastro-pleural fistula after an anastomotic gastric bypass that was managed by surgical repair without trial of an initial noninvasive approach [17].

The endoscopic intervention has become the cornerstone in managing the post-LSG gastric fistula with different modalities such as fibrin glue application, stenting, clipping, balloon dilatation, and endo-suturing [9]. It offers many advantages such as being less invasive, reducing contamination of the peritoneal cavity, saving time, and resulting in better patient recovery. The endoscopic placement of SEMS for esophageal fistula, perforation, or leak is a secure and well-established restorative method with procedure-reported mortality of around 2.2% [18]. The use of stents can improve and simplify management since stents immediately seal the leak and allow enteral diet soon after stent placement. However, the major disadvantage of the stent is that its migration rate is high, which can result in significant morbidity [19]. The Niti-S mega stent (24 mm wide, 230 mm long, with 32-mm-wide flared ends) allows insertion of the proximal end into the distal esophagus, and distally it opens into the duodenal bulb. Its larger length and diameter firmly grip the entire gastric sleeve and prevent stent migration [19]. In addition, this device has shown good efficacy in reducing the intra gastric pressure that often develop in the gastric sleeve. This mechanism contributes to the healing of the leak [20, 21]. Stent migration can also be prevented by using stents with anti-migratory cuffs [22]. In some cases, these procedures are not favorable and surgery is required. In a systemic review, done on the diagnosis and treatment options of gastric fistula in the chest after LSG, most often, there were either clips and/or clips and stents used endoscopically as the initial invasive approach. When unsuccessful, the surgical treatment was done. However, it did not mention the type of stent used that lead to failure [23].

Also in this study, a meta-analysis on endoscopic versus endoscopic and surgical treatment was done. The odds ratio (OR) of 0.526 (95% CI 0.157 to 1.757, *p* = 0.296), suggested no evidence to favor one approach over the other.

In our case, we inserted the stent successfully with no post-stenting complications.

## Conclusion

This case report highlights the complication of gastric leakage post-LSG and the morbidity associated with it. It reports the endoscopic placement of a fully covered mega Niti-S metallic stent. Endoscopic stent placement of SEMS, for the management of bariatric procedure complications, is safe and effective. A multidisciplinary approach leads to successful treatment of gastro-cutaneous and gastro-pleural fistula which is a rare entity.

## Data Availability

Can be reviewed at the editor’s request.

## Abbreviations

LSG: Laparoscopic sleeve gastrectomy
SEMS: Self-expandable metallic stents

## References

[1] Shi X (2010). A review of laparoscopic sleeve gastrectomy for morbid obesity. Obes Surg.

[2] Palermo M, Gagner M (2020). Why we think laparoscopic sleeve gastrectomy is a good operation: step-by-step technique. J Laparoendosc Adv Surg Tech.

[3] Woźniewska P, Diemieszczyk I, Hady H (2021). Complications associated with laparoscopic sleeve gastrectomy–a review. Gastroenterol Rev..

[4] Nguyen D (2016). The surgical management of complex fistulas after sleeve gastrectomy. Obes Surg.

[5] Galloro G (2015). Staple-line leak after sleve gastrectomy in obese patients: a hot topic in bariatric surgery. World J Gastrointest Endosc.

[6] Papavramidis TS, Mantzoukis K, Michalopoulos N (2011). Confronting gastrocutaneous fistulas. Ann Gastroenterol.

[7] Iqbal SM (2019). Gastropleural fistula: a rare complication of a common procedure. Cureus.

[8] Greenberg S, Kanth N, Kanth A (2015). A woman with cough: gastrobronchial fistula as a delayed complication of bariatric surgery. Case report and literature review. Am J Emerg Med.

[9] Negm S (2022). Endoscopic management of refractory leak and gastro-cutaneous fistula after laparoscopic sleeve gastrectomy: a randomized controlled trial. Surg Endosc.

[10] Anderson MJ, Sippey M (2019). Endoscopic stent placement: indications and success rates. Ann Laparosc Endosc Surg..

[11] Alvarenga ES (2016). Safety and efficacy of 1020 consecutive laparoscopic sleeve gastrectomies performed as a primary treatment modality for morbid obesity. A single-center experience from the metabolic and bariatric surgical accreditation quality and improvement program. Surg Endosc.

[12] Caiazzo R (2020). Malignant leakage after sleeve gastrectomy: endoscopic and surgical approach. Obes Surg.

[13] Alghanim F (2018). Gastropleural fistula as a rare complication of gastric sleeve surgery: a case report and comprehensive literature review. Case Rep Surg.

[14] Persaud PN (2022). A fistulous issue: gastropleural fistula as a complication of gastrectomy. Chest.

[15] Liagre A (2022). Treatment of persistent large gastrocutaneous fistulas after bariatric surgery: preliminary experience with endoscopic Kehr’s T-tube placement. Obes Surg.

[16] Gonzalez R (2007). Diagnosis and contemporary management of anastomotic leaks after gastric bypass for obesity. J Am Coll Surg.

[17] Al-Shurafa H (2017). Gastropleural fistula after single anastomosis gastric bypass. A case report and review of the literature. Int J Surg Case Rep.

[18] Ishtiaq J, Sutton J, Ahmed W (2016). A novel management of post-oesophagectomy gastro-pleural fistula. J Gastrointest Oncol.

[19] Tsai YN (2018). Endoluminal stenting for the management of leak following sleeve gastrectomy and loop duodenojejunal bypass with sleeve gastrectomy. Kaohsiung J Med Sci.

[20] Galloro G (2014). A novel dedicated endoscopic stent for staple-line leaks after laparoscopic sleeve gastrectomy: a case series. Surg Obes Relat Dis.

[21] Basha J (2014). Mega stents: a new option for management of leaks following laparoscopic sleeve gastrectomy. Endoscopy.

[22] Southwell T, Lim TH, Ogra R (2016). Endoscopic therapy for treatment of staple line leaks post-laparoscopic sleeve gastrectomy (LSG): experience from a large bariatric surgery centre in New Zealand. Obes Surg.

[23] Sakran N (2021). Gastric fistula in the chest after sleeve gastrectomy: a systematic review of diagnostic and treatment options. Obes Surg.

